# nCD64 index as a novel inflammatory indicator for the early prediction of prognosis in infectious and non-infectious inflammatory diseases: An observational study of febrile patients

**DOI:** 10.3389/fimmu.2022.905060

**Published:** 2022-07-28

**Authors:** Qianqian Liu, Yan Gao, Tao Yang, Zhe Zhou, Ke Lin, Wei Zhang, Ting Li, Yi Lu, Lingyun Shao, Wenhong Zhang

**Affiliations:** ^1^ Department of Infectious Diseases, Shanghai Key Laboratory of Infectious Diseases and Biosafety Emergency Response, National Medical Center for Infectious Diseases, Huashan Hospital, Fudan University, Shanghai, China; ^2^ Emergency Department, Huashan Hospital, Fudan University, Shanghai, China; ^3^ Life Sciences, Becton Dickinson (BD) Medical Devices (Shanghai) Co., Ltd, Shanghai, China; ^4^ Marketing, Shanghai Qianghan Medical Devices Co., Ltd, Shanghai, China; ^5^ National Clinical Research Center for Aging and Medicine, Huashan Hospital, Fudan University, Shanghai, China; ^6^ State Key Laboratory of Genetic Engineering, School of Life Science, Fudan University, Shanghai, China; ^7^ Key Laboratory of Medical Molecular Virology Key Laboratories of the Ministry of Education (MOE)/Key Laboratories of the Ministry of Health (MOH) and Institutes of Biomedical Sciences, Shanghai Medical College, Fudan University, Shanghai, China

**Keywords:** CD64, sepsis, non-infectious inflammatory diseases, infection, prognosis, hospital mortality

## Abstract

**Background:**

Generally, febrile patients admitted to the Department of Infectious Diseases, Fudan University Affiliated Huashan Hospital, China may eventually be diagnosed as infectious (ID) or non-infectious inflammatory diseases (NIID). Furthermore, mortality from sepsis remains incredibly high. Thus, early diagnosis and prognosis evaluation of sepsis is necessary. Here, we investigated neutrophil (n)CD64 index profile in a cohort of febrile patients and explored its diagnostic and prognostic value in ID and NIID.

**Methods:**

This observational cohort study enrolled 348 febrile patients from the Emergency Department and Department of Infectious Diseases. nCD64 index were detected using flow cytometry, and dynamically measured at different timepoints during follow-up. Procalcitonin (PCT), C-reactive protein (CRP), and ferritin levels were measured routinely. Finally, the diagnostic and prognostic value of nCD64 index were evaluated by receiver operating characteristic (ROC) analysis and Kaplan-Meier curve analysis.

**Results:**

Of included 348 febrile patients, 238, 81, and 29 were categorized into ID, NIID, and lymphoma groups, respectively. In ID patients, both SOFA score and infection site had impact on nCD64 index expression. In NIID patients, adult-onset Still’s disease patients had the highest nCD64 index value, however, nCD64 index couldn’t distinguish between ID and NIID. Regardless of the site of infection, nCD64 index was significantly higher in bacterial and viral infections than in fungal infections, but it could not discriminate between bacterial and viral infections. In bloodstream infections, gram-negative (G-) bacterial infections showed an obvious increase in nCD64 index compared to that of gram-positive (G+) bacterial infections. nCD64 index has the potential to be a biomarker for distinguishing between DNA and RNA virus infections. The routine measurement of nCD64 index can facilitate septic shock diagnosis and predict 28-day hospital mortality in patients with sepsis. Serial monitoring of nCD64 index in patients with sepsis is helpful for evaluating prognosis and treatment efficacy. Notably, nCD64 index is more sensitive to predict disease progression and monitor glucocorticoid treatment in patients with NIID.

**Conclusions:**

nCD64 index can be used to predict 28-day hospital mortality in patients with sepsis and to evaluate the prognosis. Serial determinations of nCD64 index can be used to predict and monitor disease progression in patients with NIID.

## Introduction

Sepsis, especially when evolving into septic shock and multiple organ dysfunction, remains a leading cause of mortality in the intensive care units (ICU) and emergency departments (EDs). Thus, the early diagnosis of sepsis, which facilitates timely and appropriate treatment, and improves the patients’ prognosis, is necessary. However, current microbiological methods, such as cultures and polymerase chain reaction (PCR), take a long time, are not sensitive enough, and it is difficult to obtain specimens. Furthermore, using common inflammatory indicators, such as neutrophil count, procalcitonin (PCT), C-reactive protein (CRP), and ferritin may make it difficult to distinguish between infectious diseases (ID) and non-infectious inflammatory diseases (NIID). Therefore, we focused on the need to address these two clinical questions: to identify novel inflammatory indicators for the early diagnosis and prognosis of sepsis, and to identify biomarkers for the rapid discrimination between ID and NIID.

CD64, a high affinity immunoglobulin (Ig)-G Fc receptor (Fcγ RI), is characterized by quick and strong inducible expression on neutrophils, in response to infections or pro-inflammatory cytokines interferon (IFN)-γ and granulocyte colony-stimulating factor (G-CSF). It was reported as an early biomarker of sepsis diagnosis in adults (1–3), neonates (4–6), and children (7), especially bacterial infections (8, 9). However, a meta-analysis reported that there are various conflicting results about the diagnosis of sepsis, which were due to different research methods and target populations (10). Furthermore, few studies have reported the profile of neutrophil (n)CD64 expression among febrile patients. Additionally, it is unclear whether the expression of nCD64 can be affected by the sites or the pathogens of infection. More importantly, in contrast with other inflammatory indicators, there is a need to evaluate whether nCD64 can distinguish between ID and NIID in predicting the prognosis and in monitoring the treatment of patients.

Accordingly, in this study, we investigated the profile of nCD64 index among febrile patients, explored the diagnostic performance of nCD64 index in the differential diagnosis of ID and NIID, and on the application of dynamic nCD64 index determination in monitoring treatment.

## Methods

### Study participants

**Figure 1:**
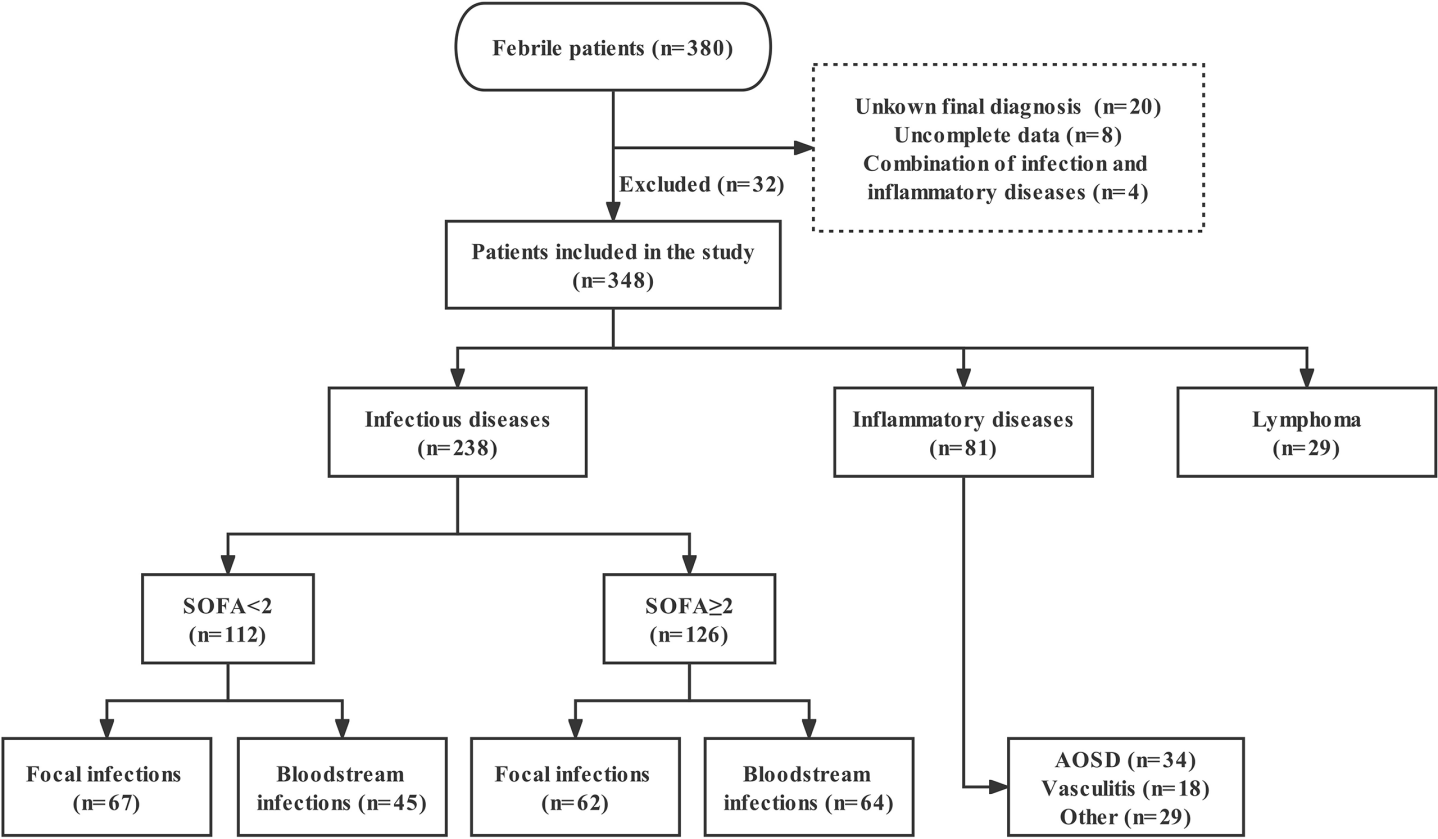
Study flowchart of patients enrollment. SOFA, Sequential Organ Failure Assessment; AOSD, adult-onset Still’s disease.

We continuously included 380 febrile (temperature >38.3°C) patients admitted to the ED or Department of Infectious Diseases at Fudan University Affiliated Huashan Hospital, China, between September 2018 and January 2020. Exclusion criteria: 1) age <18 years old; 2) human immunodeficiency virus (HIV) infection; 3) solid tumors; 4) incomplete clinical data; 5) unknown final diagnosis. In final, 32 were excluded for above reasons. According to the diagnostic criteria, the final 348 enrolled patients were divided into three groups as follows: ID, NIID, and lymphoma groups. In order to know the baseline levels of nCD64 index in healthy populations, we also recruited 23 healthy controls (HC). The distribution of patients is presented in the study flowchart (**Figure 1**).

### Ethics approval and consent to participate

This study was approved by the Ethics committee of Huashan Hospital, Fudan University (KY2021-718). Written informed consent was obtained from all participants.

### Data collection and diagnosis

At admission, the following data were recorded by clinicians for each patient: age, sex, history of immunosuppression, Sequential Organ Failure Assessment (SOFA) score, PCT, CRP, ferritin, routine laboratory results, and 28-day hospital mortality. For ID patients, SOFA score ≥2 (sepsis-3 criteria) (11) was defined as sepsis, and the site of infection was divided into focal and bloodstream infections (**Figure 1**). Septic shock refers to a state of acute circulatory failure characterized by persistent arterial hypotension (12). The etiological examination included specimen (blood, sputum, urine, etc.) culture, next-generation sequencing, PCR and so on. ID patients without etiology were clinically diagnosed based on symptoms, laboratory test, imaging, and effective anti-infection therapy. Those in the NIID group included patients with adult-onset Still’s disease (AOSD), vasculitis, and other inflammatory disorders (such as systemic lupus erythematosus, rheumatoid arthritis, dermatomyositis, and connective tissue diseases). Notely, AOSD remains a diagnosis of exclusion, is defined based on Yamaguchi criteria, highlighting the necessity to rule out infectious, autoimmune or malignant diseases (13). The diagnosis of lymphoma was based on the histopathological examination. In addition, diagnosis of hemophagocytic lymphohistocytosis (HLH) requires 5 out of 8 HLH-2004 criteria to be fulfilled (14).

### Measurement of nCD64 index

Peripheral ethylenediaminetertraacetic acid (EDTA) anticoagulated blood samples were collected from enrolled individuals and processed within 24 h at room temperature. Whole blood (50 μL) samples were labelled with 30 μL of antibody mixture, consisting of equal quantities of CD45 PerCP (clone: 2D1), CD14 fluorescein isothiocyanate (FITC) (clone: MΦP9) and CD64 PE (clone: 10.1) (BD Bioscience, San Jose, CA, USA). After 15 min incubation, red blood cells were lysed with lysing solution (BD Bioscience, San Jose, CA, USA), and 10 min after, were washed twice. Finally, the expressions of CD64 were measured using flow cytometry (FACSCanto plus, BD Bioscience), presented as median fluorescence intensity (MFI), and the data were analyzed using FlowJo™ Software 10 (Ashland, OR: Becton, Dickinson, and Company). The formula for calculating the nCD64 index is shown below: nCD64 index = (nCD64 MFI/lymCD64 MFI)/(mCD64 MFI/nCD64 MFI), where n, lym and m represent neutrophils, lymphocytes and monocytes, respectively. In addition, the qualification of stability of the nCD64 index in stored blood samples and method details were described in our previous research (15).

### Statistical analysis

Statistical analysis was performed using GraphPad Prism 8 (GraphPad, Inc., CA, USA). Continuous variables are presented as median with interquartile range (IQR) and categorical variables as number and percentage. The comparisons of continuous variables between independent groups were performed using Mann-Whitney U test (two groups), and Kruskal-Wallis test (multiple groups) followed by Dunn’s post-test for multiple comparisons. Categorical variables were compared using χ^2^ test or Fisher’s exact test, as appropriate. For the correlations, Spearman correlation coefficient was used. Receiver operating characteristics (ROC) curve analysis was performed to predict hospital death within 28 days in patients with ID. Kaplan-Meier curve analysis was performed to calculate the survival rate at 28 days for ID patients, and compared using log-rank (Mantel-Cox) test. An adjusted *P* value of less than 0.05 was considered significant.

## Results

### Characteristics of the patients

The baseline characteristics of enrolled patients, compared according to ID and NIID groups, are summarized in **Table 1**. Of 348 enrolled febrile patients, 238 (68.39%), 81 (23.28%), and 29 (8.33%) had ID, NIID, and lymphoma, respectively. Of 238 ID group patients, those with sepsis (SOFA score≥2, n = 126) were divided into focal infections (n = 62; of whom 46, 13, 2, and 1 patient had pneumonia, urinary tract infection [UTI], biliary infection, and pericardial effusion, respectively) and bloodstream infections (n = 64; of whom 53 and 11 had bacterial and viral infections, respectively) groups. Of 112 infected patients without sepsis (SOFA score<2), 67 had focal infections (of whom 40, 15, 8, 2, 1, and 1, had pneumonia, UTI, liver abscess, celiac infection, skin infection, and enteritis) and 45 had bloodstream infection (of whom 3 and 42 had infective endocarditis and microbiologically-confirmed diagnoses), respectively. Of 81 NIID patients, 34 were AOSD patients, 18 were vasculitis, and the remaining 29 were classified as other.

**Table 1:** Baseline characteristics of enrolled patients and controls.

Characteristics	Febrile patients (n = 348)			Controls (n = 23)	^1^ *P* value
**Group**	ID (n = 238)	NIID (n = 81)	Lymphoma (n = 29)	HC (n = 23)	NA
**Age, y**	64 (49-73)	49 (30-64)	51 (29-66)	28 (24-51)	<0.0001
**Male, n (%)**	163 (68.50)	27 (33.30)	15 (51.70)	5 (21.70)	<0.0001
**History of immunosuppression, n (%)**	25 (10.50)	10 (12.35)	4 (13.79)	NA	ns
**SOFA score**	2 (0-4)	NA	NA	NA	NA
**WBC, ×10^9 cells/L**	8.00 (5.39-11.32)	9.83 (5.86-17.15)	3.79 (1.99-7.25)	NA	0.0391
**Neutrophil, %**	77.85 (67.45-87.80)	79.70 (68.58-88.45)	65.50 (54.75-82.25)	NA	ns
**Monocyte, %**	6.30 (4.13-8.83)	5.40 (3.43-8.78)	10.75 (5.43-13.80)	NA	ns
**Lymphocyte, %**	12.70 (5.75-22.10)	12.00 (6.50-18.90)	17.90 (8.35-25.00)	NA	ns
**PCT, ng/mL**	0.37 (0.10-2.33)	0.16 (0.06-0.41)	0.43 (0.15-0.67)	NA	0.0002
**CRP, mg/L**	58.25 (15.15-142.30)	46.50 (14.10-136.00)	46.15 (18.28-117.50)	NA	ns
**Ferritin, ng/mL**	887 (399-1817)	1449 (606-2000)	2000 (1607-2000)	NA	0.0090
**nCD64 index**	15.82 (3.30-57.16)	23.16 (7.45-50.58)	87.02 (30.36-221.8)	0.83 (0.42-1.15)	ns
**28-day mortality, n (%)**	22 (9.24)	1 (1.23)	2 (6.90)	NA	0.0125

Categorical variables are shown as number and percentage, n (%); and continuous variables as median with interquartile range (IQR), M (IQR). The comparisons of continuous variables between ID and NIID groups were performed using Mann-Whitney U test. Categorical variables were compared using χ^2^ test or Fisher’s exact test, as appropriate.

^1^ Compared between ID and NIID; ID, infectious diseases; NIID, non-infectious inflammatory diseases; HC, healthy control; NA, not available; ns, not significant; SOFA, Sequential Organ Failure Assessment; WBC, white blood cell; PCT, procalcitonin; CRP, C-reactive protein; nCD64, neutrophil CD64.

### Comparison of nCD64 index among patients with febrile diseases

**Figure 2:**
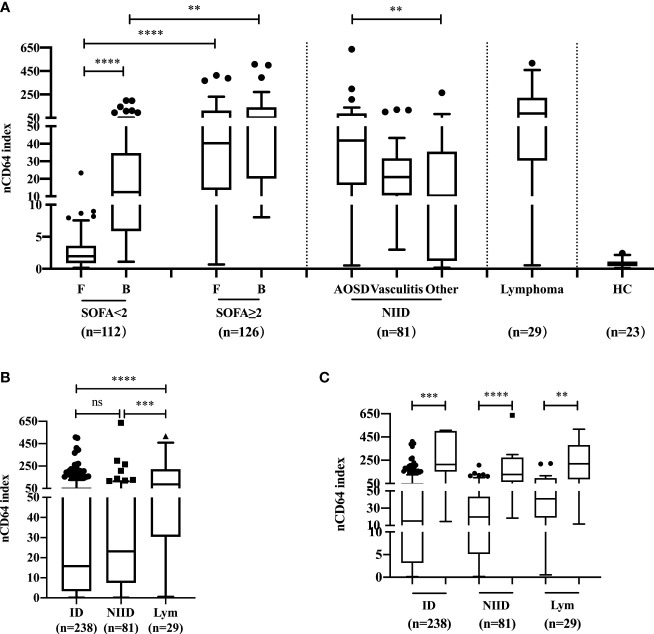
The profile and comparison of nCD64 index among patients with febrile diseases. **(A)** The investigation of nCD64 index in febrile patients and HC. **(B)** The difference of nCD64 index among ID, NIID and lymphoma. **(C)** The profile of nCD64 index during hemophagocytic lymphohistocytosis induced by various etiologies, the circle (left) represents the patients with hemophagocytic lymphohistocytosis and the square (right) represents those without hemophagocytic lymphohistocytosis. The data is presented as median with interquartile range (IQR). The comparisons of nCD64 index between independent groups were performed using Mann-Whitney U test (two groups), and Kruskal-Wallis test (multiple groups) followed by Dunn’s post-test for multiple comparisons. **P<0.01, ***P<0.001, **** *P*<0.0001. nCD64, neutrophil CD64; F, focal infection; B, bloodstream infection; SOFA, Sequential Organ Failure Assessment; AOSD, adult-onset Still’s disease; HC, healthy controls; ID, infectious diseases; NIID, non-infectious inflammatory diseases; Lym, lymphoma; IQR, interquartile range; ns, no significance.

The profiles of nCD64 expression among febrile patients among ID, NIID, and lymphoma groups based on the final diagnoses are shown in **Figure 2**. Among patients with no sepsis (SOFA score<2), those with bloodstream infection had a higher nCD64 index than the patients with focal infection (12.37 vs 1.96, *P*<0.0001), while having SOFA score ≥2 showed no significant difference in nCD64 index between bloodstream and focal infection groups (F: 40.32 *vs* B: 52.12, *P*=0.7868) (**Figure 2A**). nCD64 index was higher in patients with sepsis (SOFA≥2) than in patients without sepsis (SOFA<2) regardless of the site of infection (F: 40.32 vs 1.96, *P*<0.0001; B: 52.12 *vs* 12.37, *P*=0.0017, **Figure 2A**). Regarding the NIID group, nCD64 index was highest in patients with AOSD, and four times higher in other NIID patient group (41.86 vs 10.33, *P*=0.0035), besides the vasculitis patient group. Patients with lymphoma exhibited the highest level of nCD64 index among febrile groups (**Figures 2A, B** and **Table 1**); but with no significant difference between ID and NIID groups (15.82 *vs* 23.16, *P*>0.99) (**Figure 2B** and **Table 1**). The nCD64 index increased 10-fold when febrile patients developed HLH, regardless of the etiology of HLH such as ID, NIID, or lymphoma (**Figure 2C**).

### nCD64 index in distinguishing between different infectious pathogens

**Figure 3:**
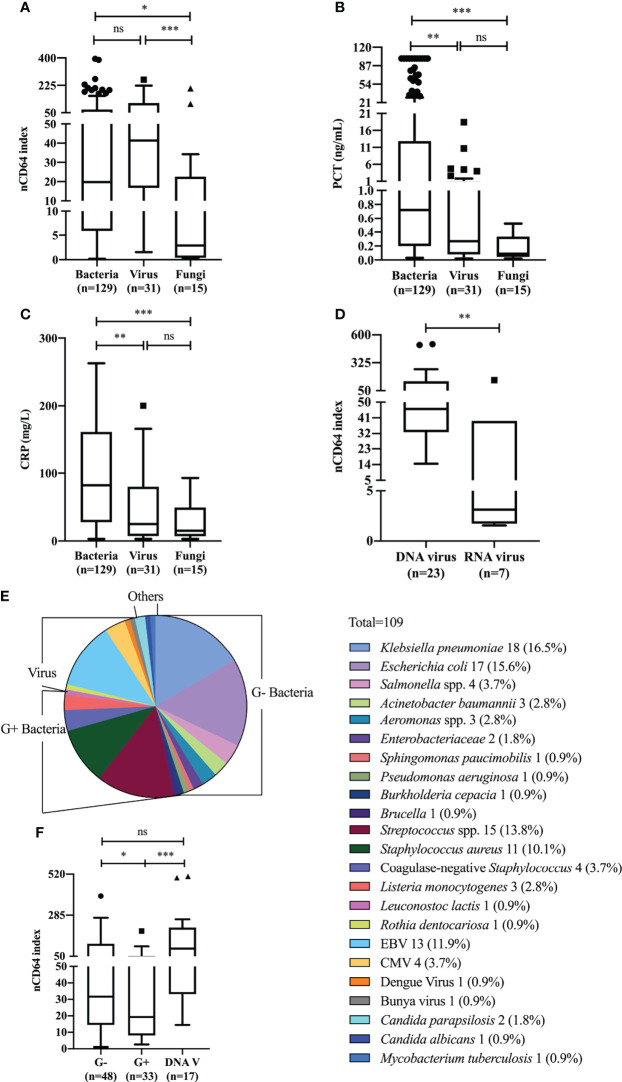
nCD64 index of different pathogens in patients with sepsis. Differences of nCD64 index **(A)**, PCT **(B)**, and CRP **(C)** in bacterial, viral and fungal infections. **(D)** The comparison of nCD64 index in DNA and RNA viral infections. **(E)** Microbial composition of blood samples in patients with bloodstream infections. **(F)** The comparison of nCD64 index in G-, G+ bacterial and DNA viral bloodstream infections. The comparisons of nCD64 index, PCT and CRP between independent groups were performed using Mann-Whitney U test (two groups), and Kruskal-Wallis test (multiple groups) followed by Dunn’s post-test for multiple comparisons. *P<0.05, **P<0.01, ***P<0.001. nCD64, neutrophil CD64; PCT, procalcitonin; CRP, C-reactive protein; DNAV, DNA virus; EBV, Epstein Barr virus; CMV, cytomegalovirus; ns, no significance.

We next analyzed whether nCD64 index could be used to differentiate between bacterial (n = 129), viral (n = 31) and fungal (n = 15) infections. nCD64 index increased in bacterial (19.78 *vs* 2.90, *P*=0.0166) (n=129) and viral (41.38 *vs* 2.90, *P*=0.0005) infections compared with fungal infections. No significant difference of nCD64 index occurred between bacterial and viral infections (19.78 *vs* 41.38, *P*=0.0916) (**Figure 3A**), which is different from that of common inflammatory indicators, with both PCT and CRP levels being higher in patients with bacterial infections than in those with viral and fungal infections (**Figures 3B, C**). When nCD64 index was compared between DNA (n=23) and RNA viral (n=7) infections, more than 10-fold increase in nCD64 index was found in DNA compared with RNA viral infections (46.14 *vs* 3.13, *P*=0.0071) (**Figure 3D**). The pathogen profile of 106 patients with bloodstream infections presented in **Figure 3E** revealed that 45.28% and 31.13% were G- and G+ bacteria, respectively, and viruses and others accounted for 17.93% and 5.66%, respectively (**Table 2**). Infection was polymicrobial in four patients. nCD64 index in patients with G- bacterial (31.65 *vs* 19.33, *P*=0.0394) and DNA viral infections (93.73 *vs* 19.33, *P*=0.0009) were significantly higher than those in patients with G+ bacterial infections, with no significant difference in nCD64 index between G- bacterial and DNA viral infections (31.65 *vs* 93.73, *P*=0.1984) (**Figure 3F**).

**Table 2:** Pathogens of bloodstream infections.

Pathogen	Number (%)	nCD64 index, M (IQR)
**G+ bacteria**	48 (45.28)	19.33 (8.11-51.92)
**G- bacteria**	33 (31.13)	31.65 (14.47-121.80)
**^1^ DNA virus**	17 (16.04)	93.73 (33.17-214.10)
**^2^ RNA virus**	2 (1.89)	7.55 (1.73-13.36)
***Mycobacterium tuberculosis***	1 (0.94)	197.60
**Fungi**	3 (2.83)	34.25 (22.57-105.70)
**G- and G+ bacteria**	2 (1.89)	19.58 (13.63-25.53)

^1^ included EBV (n = 13) and CMV (n = 4).

^2^ included Dengue virus (n = 1) and Bunya virus (n = 1).

IQR, interquartile range; G-, Gram negative; G+, Gram positive.

### The correlation between severity of sepsis and nCD64 index

**Figure 4:**
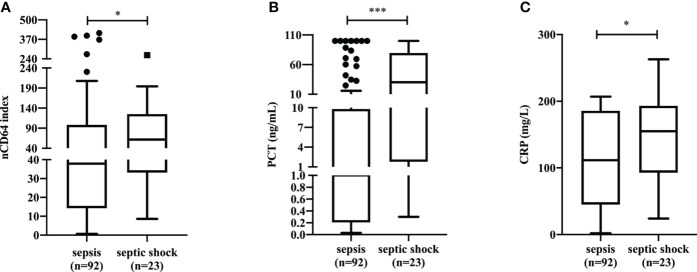
The differences of nCD64 index **(A)**, PCT **(B)**, and CRP **(C)** in sepsis patients with and without shock. The comparisons of nCD64 index, PCT and CRP between two groups were performed using Mann-Whitney U test. *P<0.05, ***P<0.001. nCD64, neutrophil CD64; PCT, procalcitonin; CRP, C-reactive protein; ns, no significance.

Among 126 patients with sepsis (SOFA≥2), the 23 (18.25%) that experienced septic shock, had a significantly higher nCD64 index value than that of patients without septic shock (61.94 *vs.* 37.94, *P*=0.0446) (**Figure 4A**). Patients with septic shock also presented with higher levels of traditional inflammatory indicators including PCT (30.22 ng/mL *vs* 1.00 ng/mL, *P*=0.0002) (**Figure 4B**) and CRP (155.0 mg/L *vs.* 111.5 mg/L, *P*=0.0436) (**Figure 4C**) than those in patients without septic shock. Furthermore, we found a moderately positive correlation between nCD64 index and SOFA score, PCT, and CRP (**Figure S1** in Additional file 1).

### nCD64 index and prognosis of patients with infection

**Figure 5:**
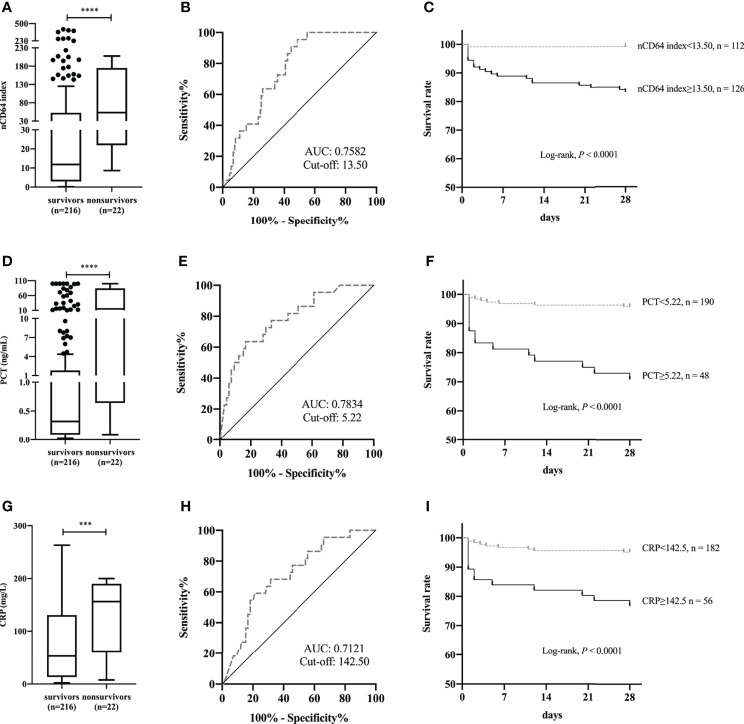
Comparison of nCD64 index **(A)**, PCT **(D)**, and CRP **(G)** between 28-day hospital survivors and non-survivors. The ROC curves analysis of nCD64 index **(B)**, PCT **(E)**, and CRP **(H)** for predicting 28-day hospital death. Kaplan-Meier survival curves for patients with the best cut-off value of nCD64 index **(C)**, PCT **(F)**, and CRP **(I)**, compared using log-rank test. ***P<0.001, ****P<0.0001. nCD64, neutrophil CD64; PCT, procalcitonin; CRP, C-reactive protein; ROC, receiver operating characteristics; AUC, area under the curve.

To further test the performance of nCD64 index on the prognosis of patients with infection, 238 patients in the ID group were classified according to whether they died within 28 days or not; 22 (9.24%) died within 28 days (non-survivors). Non-survivors showed a significant increase in nCD64 index compared with survivors (53.28 *vs* 11.81, *P*<0.0001) (**Figure 5A**). ROC curve analysis established 13.50 as the best cut-off value to identify 28-day hospital mortality, with a sensitivity of 95.45% (95% confidence interval [CI], 78.20%-99.77%) and specificity of 51.39% (95% CI, 44.76%-57.97%) (**Figure 5B**). Patients with nCD64 index≥13.50 had a greater 28-day hospital mortality compared to patients with nCD64 index<13.50, according to Kaplan-Meier survival curve (16.67% *vs* 0.89%, *P*<0.0001) (**Figure 5C**). Similarly, non-survivors presented with higher levels of PCT and CRP than survivors (**Figures 5D, G**). PCT>5.22 ng/mL or CRP>142.50 mg/L at admission, the best cut-off by ROC curve analysis, predicted 28-day hospital mortality with sensitivity of 63.64% (95% CI, 42.95%-80.27%) *vs*. 59.09% (95% CI, 38.73%-76.74%) and specificity of 83.33% (95% CI, 77.61%-87.82%) *vs*. 78.92% (95% CI, 72.82%-83.96%) (**Figures 5E, H**). Patients with PCT≥5.22 ng/mL had a lower 28-day survival rate compared to patients with PCT<5.22 ng/mL (70.83% *vs.* 95.79%, *P*<0.0001) (**Figure 5F**). Patients with CRP≥142.50 mg/L had a lower 28-day survival rate compared to patients with CRP<142.50 mg/L (76.79% *vs.* 95.06%, *P*<0.0001) (**Figure 5I**).

### Serial determinations of nCD64 index in patients with sepsis and NIID

**Figure 6:**
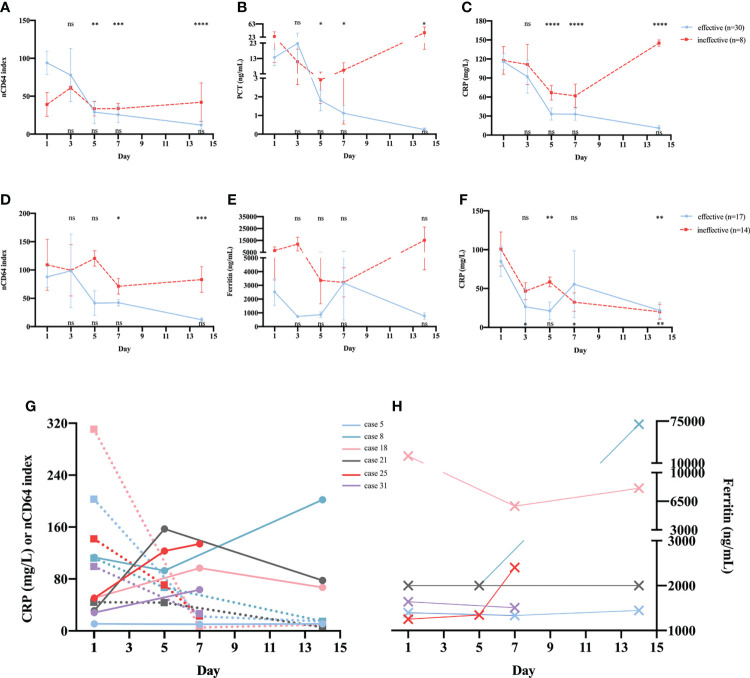
Serial determinations of nCD64 index during treatment in patients with sepsis or NIID. Dynamic changes of nCD64 index **(A)**, PCT **(B)**, and CRP **(C)** over time in patients with sepsis. Dynamic changes of nCD64 index **(D)**, PCT **(E)**, and CRP **(F)** over time in patients with NIID. Blue lines represent patients who received effective treatment, and red lines represent patients who received ineffective treatment. The comparisons were performed between follow-up data and baseline data. Data were analyzed using Mann-Whitney U test. The results of statistical analysis in effective and ineffective treatment group were shown at the top and bottom of graph, respectively. **(G, H)** The trends of nCD64 index, CRP and ferritin over time among six cases in the ineffective treatment NIID group, the dashed lines represent CRP, and the solid lines represent nCD64 index. *P<0.05, **P<0.01, ***P<0.001, ****P<0.0001. nCD64, neutrophil CD64; CRP, C-reactive protein; NIID, non-infectious inflammatory diseases; ns, no significance.

The 38 patients with sepsis in whom serial determination of nCD64 index was performed were divided into two groups according to whether the treatment was effective or not; 30 patients were in the effective treatment group. Patients who received effective antibiotic treatment had reduced nCD64 index on day 5 and persistent decrease in nCD64 index over time (**Figure 6A**, blue line); this was consistent with the PCT and CRP trends (**Figures 6B, C**, blue line). However, in the ineffective treatment group (n=8), the nCD64 index was at a high level without an evident decrease over time, like the PCT and CRP levels (**Figures 6A–C**, red line).

Of the 31 patients with NIID in whom serial determination of nCD64 index was performed, 17 and 14 were in the effective and ineffective treatment groups, respectively. As shown in (**Figure 6D**, patients who received effective glucocorticoid treatment had reduced nCD64 index on day 7 and persistent decrease over time, which differed from those of common inflammatory indicators, including ferritin (**Figure 6E**) and CRP (**Figure 6F**). More importantly, in the ineffective treatment group, both nCD64 index and ferritin were persistently elevated over time; however, the level of CRP decreased on day 7. In addition, we further analyzed the details of six cases in the ineffective treatment group who presented with different trends in nCD64 index, CRP, and ferritin over time (**Table S1** in Additional file 1). We observed that the time when the patients’ condition was exacerbated was consistent with the time when nCD64 index increased, which was prior to the time when ferritin and CRP levels changed (**Figures 6G, H**; **Table S1** in Additional file 1).

## Discussion

Although the high expression of CD64 on neutrophil has been proposed as a promising biomarker to diagnose sepsis at admission in the last several years, few studies have explored the profile of nCD64 index according to sites and pathogens of infection in patients with sepsis. Our cohort of patients in the present study, including ID, NIID, and lymphoma patients, reflected the real-life clinical spectrum of febrile patients at the general tertiary hospital in which nCD64 index could potentially be applied.

We observed that the nCD64 index was the highest in patients with lymphoma compared with the index in the other two groups; indicating that it is necessary to rule out the diagnosis of lymphoma when making use of nCD64 index to diagnose infections. Moreover, there was no significant difference between ID and NIID groups in nCD64 index, suggesting that nCD64 index could not be used to distinguish between ID and NIID differential diagnoses. These results are contradictory to those of previous research in patients with inflammatory diseases (16–18), which reported that CD64 expression on neutrophil had a good performance in discriminating between ID and NIID. These conflicting results are potentially due to the following reasons: in our study, the NIID cohort included patients with AOSD, who presented with a significantly higher level of nCD64 index than that in other NIID groups. This resulted in the median nCD64 index in the NIID group reaching similar level to that of ID group. Most previous studies focused on the value of nCD64 in the early diagnosis of bacterial infections or sepsis; therefore, we further explored the profile of nCD64 index in patients with ID classified according to the site of infection and SOFA score. Notably, our results showed a higher nCD64 index in patients with bloodstream infection than that in patients with focal infection when SOFA score was <2; however, the level of nCD64 index did not seem to be affected by the site of infection in patients with sepsis (SOFA≥2). As expected, with the same infection site, the nCD64 index in patients with sepsis (SOFA≥2) showed a 4- to 20-fold increase higher than that in patients without sepsis (SOFA<2). This result suggest that a correlation exists between the high nCD64 index level and severe sepsis. As we reported in a previous study on chronic *Mycobacterium tuberculosis* infection, the nCD64 index as an inflammatory indicator, reflects the inflammatory cascade response, and presents a high feasibility of use in daily practice (15). Infection, autoimmune disorder, and hematologic malignancy can induce secondary HLH, also known as macrophage activation syndrome (MAS), which leads to an increase in the risk of early death (19). Therefore, we compared the level of nCD64 index in patients with and without HLH. Although there was no difference in the nCD64 index among patients with HLH induced by various etiologies including ID, NIID, and lymphoma, the nCD64 index of patients with HLH increased significantly 10-fold, compared to that of patients without HLH. This result might be due to the uncontrolled production of IFN-γ or other pro-inflammatory cytokines in HLH, which strongly induced the expression of CD64 on neutrophil.

The median of nCD64 index could be at least six times higher in patients with bacterial and viral infections than that in patients with fungal infection; however, nCD64 index did not differ significantly between bacterial and viral infections, unlike PCT and CRP. The surprising difference between our results and the reported literature may be explained by several reasons. First, as observed in a previous study nCD64 was distinct between DNA and RNA viral infections, with sensitivity of 90.5% and specificity of 81.8% (16). Most viral infections in our study were DNA viral infections (n = 23, 74.19%), presenting with an extremely high nCD64 index value, similar to that in G- bacterial infections. Second, of the 23 patients with DNA viral infections, about half (n = 14) had Epstein Barr virus infections, and 5 were accompanied by HLH (21.74%). In these five, excessive inflammatory response resulted in a high nCD64 index value (20); however, there were no patients with HLH in the bacterial infection group. Third, in the present study, the composition of infection sites among bacterial, viral, and fungal infections differed (**Figure S2** in Additional file 1); the proportion of bloodstream infections in bacterial (63.6%) and viral (61.3%) infections was much higher than that in fungal (20.0%) infections. In order to control for the confounding factor because of the different infection sites, we focused on patients with bloodstream infections whose pathogens were classified into G- bacteria, G+ bacteria, viruses, and others. Notably, compared with G+ bacterial infections, both DNA viral and G- bacterial infections showed a much higher level of nCD64 index. In agreement with previous studies (16, 21), G- bacteria induced a higher nCD64 expression than G+ bacteria. Unlike previous studies on critically ill patients (22, 23), patients with G- and G+ bacterial infections had similar nCD64 expression, without distinguishing between the sites of infection. Moreover, Xiong et al. observed in febrile patients in a hematology department that no significant variation occurred in nCD64 index among G-, G+ bacterial, and fungal infections, in positive blood cultures.

In patients with septic shock, nCD64 index was almost twice as high as that in sepsis patients without shock. Similarly, the commonly used biomarkers of infection, such as PCT and CRP, presented with higher levels in patients with septic shock, as compared to those without septic shock (22). Although a moderate or weak correlation was found between nCD64 index and SOFA, PCT, and CRP, which suggested that nCD64 index might be a useful marker for predicting disease severity in sepsis, it should be noted that nCD64 index is not a substitute for these commonly used indicators of infection (23). Our data illustrated that non-survivor patients showed increased levels of nCD64 index at admission compared to survivors. Concerning the performance of nCD64 index in predicting the 28-day hospital mortality among patients with infection, the AUC (0.7582), at the cut-off value of 13.50, was higher than that of CRP (0.7121), but lower than that of PCT (0.7834). Differentially, compared with PCT (sensitivity: 63.64%, specificity: 83.33%), nCD64 index was characterized by high sensitivity (95.45%) and low specificity (51.39%). Therefore, further research combining these biomarkers should be performed to confirm the utility and clinical application (2).

As reported earlier, patients with infection who received effective antibiotics treatment showed a continuous reduction in nCD64 index over time (23). Our results indicated that patients with sepsis who received effective therapy showed direct relationship with decreased nCD64 index on day 5, and continuous decrease over time. Conversely, the ineffective antibiotics treatment group persistently had a high level of nCD64 index within 2 weeks of dynamic monitoring. Similar to those of several previous studies (24, 25), with the improvement in the clinical condition, sepsis patients treated with appropriate therapy had a quick reduction of nCD64 index. Although a meta-analysis (2) illustrated that nCD64 showed a diagnostic performance for sepsis superior to CRP and PCT regarding the monitoring of antibiotics treatment, our findings suggest that nCD64 index had an equivalent value to CRP and PCT. To our knowledge, the present study is the first to address the time course of nCD64 index in relation to the effective treatment with glucocorticoids in patients with NIID. Patients with NIID treated with appropriate glucocorticoids had a significant decrease in day-7 nCD64 index. In contrast, NIID patients who were insensitive or unresponsive to glucocorticoids exhibited no significant reduction in day-7 nCD64 index, and had an almost 2-fold value of nCD64 index compared to those that received effective glucocorticoids treatment. At present, CRP and ferritin are widely used in routine laboratory tests to evaluate treatment with glucocorticoids in patients with NIID. However, our data showed that CRP and ferritin were not sensitive when reflecting the diseases progression, regardless of the effect of glucocorticoids treatment. Notably, in our study, nCD64 index was observed to have a predictive value in evaluating the exacerbation of NIID conditions (**Table S1** in Additional file 1). Moreover, we found that the dynamic detection of nCD64 index could guide glucocorticoids therapy uses in patients with NIID.

Our study had several limitations. First, the present investigation was a single-center research, which restricted the universal application of our result due to different spectrum of febrile diseases. Second, the number of RNA viral and fungal infections in patients with bloodstream infection in our study was relatively small, which might cause a certain degree of bias. Third, the clinical application value of nCD64 index was not compared with those of interleukin (IL)-6 and IL-18, which have been reported more in recent years (26). Furthermore, in the future, we will further explore the functions of nCD64 in the process of sepsis induced by infections and the mechanisms of nCD64 in inflammatory regulation.

In our study, we investigated in detail the expression of nCD64 index in febrile patients with different disease spectrums. In patients with infection, both SOFA score and the site of infection had impact on the expression of nCD64 index. In patients with NIID, those with AOSD had the highest value of nCD64 index. In addition, the nCD64 index could not be used to distinguish between ID and NIID patients. Regardless of the site of infection, the nCD64 index was significantly higher in bacterial and viral infections than in fungal infections, but it could not be used to discriminate between bacterial and viral infections. In bloodstream infections, G- bacterial infections showed an obvious increase in nCD64 index compared with G+ bacterial infections. More importantly, the nCD64 index has the potential to be a biomarker for distinguishing DNA and RNA viral infections. The routine measurement of nCD64 index can facilitate septic shock diagnosis and predict 28-day hospital mortality in patients with sepsis. Moreover, serial monitoring of nCD64 index in patients with sepsis is helpful to evaluate the prognosis and treatment efficacy. Notably, the nCD64 index is more sensitive to predict disease progression and to monitor glucocorticoid treatment in patients with NIID.

## Data availability statement

The original contributions presented in the study are included in the article/**Supplementary Material**. Further inquiries can be directed to the corresponding authors.

## Ethics statement

The study involving human participants was reviewed and approved by the Ethics committee of Huashan Hospital, Fudan University (KY2021-718). Written informed consent was obtained from all participants. The patients/participants provided their written informed consent to participate in this study.

## Author contributions

QL and YG: Critical analysis, interpretation of the data, and drafting of the manuscript. TY and WeiZ: Sample processing and cohort management. ZZ and KL: Clinical data collection and sample acquisition. TL and YL: Guidance of experiment and technical support. LS and WenZ: Conceived and designed the study. All authors contributed to the article and approved the final version of the manuscript.

## Funding

The study was partly financed by the Key Laboratory Project of Shanghai Science and Technology Commission (grant number 20dz2210401 and 20dz2210402), and Shanghai Sailing Program (grant number 21YF1404200). The technical support was partly made by the BD Medical Devices (Shanghai) Co., Ltd. The funders had no role in study design, data collection, analysis and interpretation, writing and submission of the manuscript.

## Acknowledgments

We thank all the individuals for their participation in this study.

## Conflict of interest

Author TL was employed by BD Medical Devices (Shanghai) Co., Ltd. Author YL was employed by Shanghai Qianghan Medical Devices Co., Ltd.

The remaining authors declare that the research was conducted in the absence of any commercial or financial relationships that could be construed as a potential conflict of interest.

## Publisher’s note

All claims expressed in this article are solely those of the authors and do not necessarily represent those of their affiliated organizations, or those of the publisher, the editors and the reviewers. Any product that may be evaluated in this article, or claim that may be made by its manufacturer, is not guaranteed or endorsed by the publisher.
