# Supplementary material for: nCD64 index as a novel inflammatory indicator for the early prediction of prognosis in infectious and non-infectious inflammatory diseases: An observational study of febrile patients
Source: Front Immunol. 2022 Jul 28;13:905060. doi: 10.3389/fimmu.2022.905060 (PMC9367970; doi:10.3389/fimmu.2022.905060)
Supplement: Supplementary file 1 [file DataSheet_1.docx]

**Supplementary materials**

**
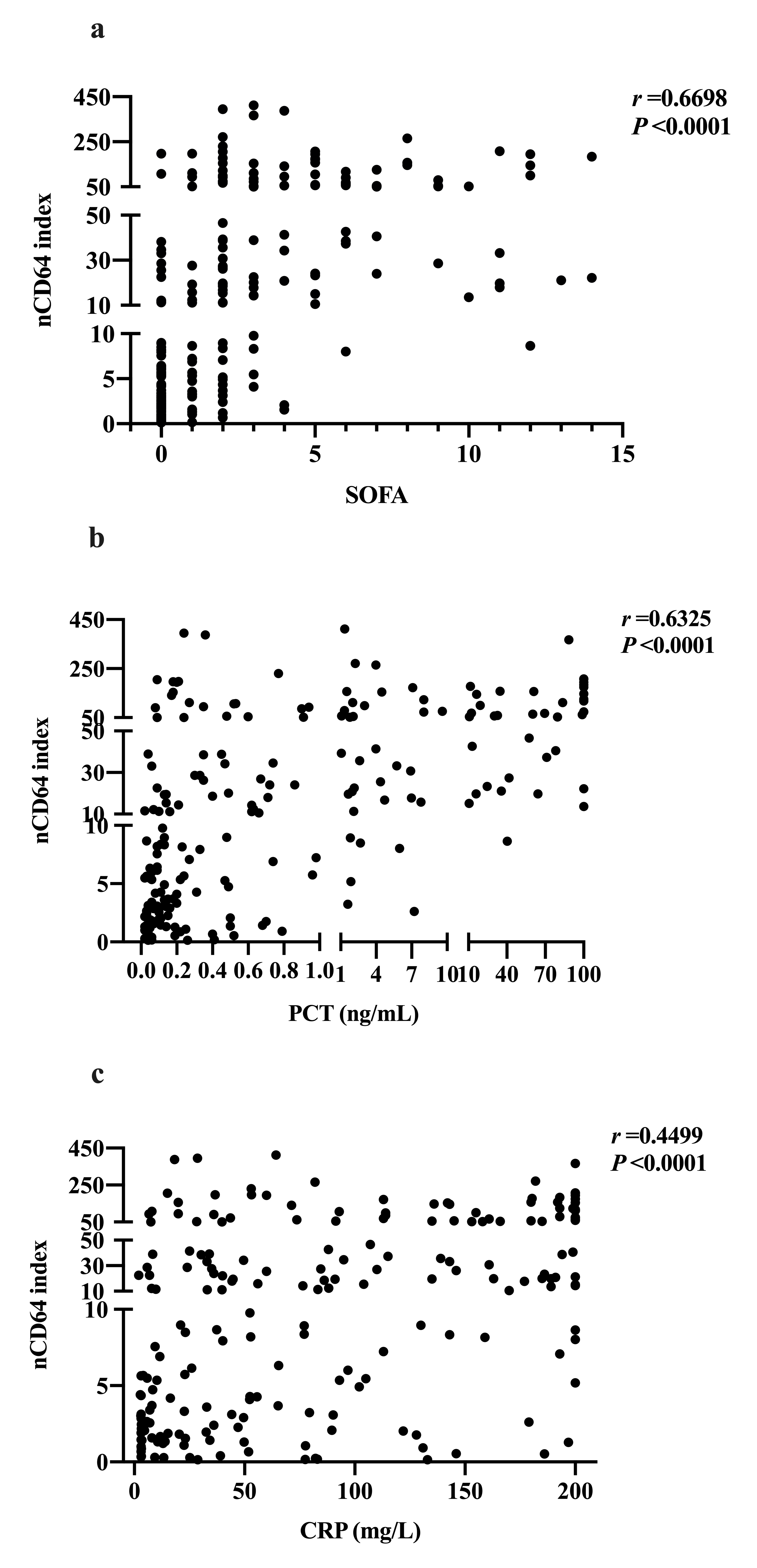
**

**Figure S1.** The correlation between nCD64 index and SOFA (a), PCT (b) and CRP (c).

**Figure S2.** The composition of infection sites in bacterial, viral and fungal infections. BSI, bloodstream infection.

**Table S1. The dynamics of nCD64 index in patients with non-infectious inflammatory diseases.**

| Case | Diagnosis | nCD64 index | | | CRP (mg/L) | | | Ferritin (ng/mL) | | | Disease description |
| --- | --- | --- | --- | --- | --- | --- | --- | --- | --- | --- | --- |
|  |  | T1 | T2 | T3 | T1 | T2 | T3 | T1 | T2 | T3 |  |
| Case 5 | Vasculitis | 10.89 | 10.21 | 10.87 | 203.00 | 22.20 | 14.50 | 1395 | 1336 | 1447 | Progressive enlargement of lung lesions |
| Case 8 | AOSD | 113.41 | 92.85 | 202.25 | 112.00 | 66.80 | 14.70 | >2000 | >2000 | 70192 | Hemophagocyic lymphohistiocytosis |
| Case 18 | AOSD | 50.71 | 96.85 | 67.12 | 310.81 | 5.33 | 9.51 | 20893 | 5904 | 8080 | Hemophagocyic lymphohistiocytosis |
| Case 21 | AOSD | 31.21 | 157.28 | 77.84 | 44.30 | 43.50 | 5.83 | >2000 | >2000 | >2000 | Progressive elevation of liver enzyme |
| Case 25 | AOSD | 50.45 | 123.27 | 134.20 | 142.00 | 70.70 | 23.20 | 1252 | 1344 | 2403 | Poor effectiveness of glucocorticoid |
| Case 31 | AOSD | 28.06 | 63.61 | - | 99.30 | 26.40 | - | 1642 | 1508 | - | Aggravated rash |

Abbreviations: nCD64, neutrophil CD64; CRP, C-reactive protein; T, timepoint; AOSD, Adult onset Still’s disease.
